# Loss in Pluripotency Markers in Mesenchymal Stem Cells upon Infection with *Chlamydia trachomatis*

**DOI:** 10.4014/jmb.2406.06023

**Published:** 2024-12-07

**Authors:** Munir A. Al-Zeer, Mohammad Abu Lubad

**Affiliations:** 1 Department of Biological Sciences, School of Science, The University of Jordan, Amman 11942, Jordan; 2 Microbiology and Immunology Department, Faculty of Medicine, Mutah University, Al-Karak, Jordan

**Keywords:** Mesenchymal stem cells, pluripotent cells, loss of stemness markers, *Chlamydia trachomatis*

## Abstract

The intracellular pathogen *Chlamydia trachomatis* can inflict substantial damage on the host. Notably, *Chlamydia* infection is acknowledged for its precise modulation of diverse host signaling pathways to ensure cell survival, a phenomenon intricately connected to genetic regulatory changes in host cells. To monitor shifts in gene regulation within *Chlamydia*-infected cells, we employed mesenchymal stem cells (MSCs) as a naïve, primary cell model. Utilizing biochemical methods and imaging, our study discloses that acute *Chlamydia* infection in human MSCs leads to the downregulation of transcription factors Oct4, Sox2, and Nanog, suggesting a loss of pluripotency markers. Conversely, pluripotency markers in MSCs were sustained through treatment with conditioned medium from infected MSCs. Additionally, there is an augmentation in alkaline phosphatase activity, along with elevated Sox9 and CD44 mRNA expression levels observed during acute infection. A comprehensive screening for specific cell markers using touchdown PCR indicates an upregulation of mRNA for the early chondrogenesis gene Sox9 and a decrease in mRNA for the MSC marker vimentin. Real-time PCR quantification further corroborates alterations in gene expression, encompassing increased Sox9 and CD44 mRNA levels, alongside heightened alkaline phosphatase activity. In summary, the infection of MSCs with *C. trachomatis* induces numerous genetic deregulations, implying a potential trend towards differentiation into chondrocytes. These findings collectively underscore a targeted impact of *Chlamydia* on the gene regulations of host cells, carrying significant implications for the final fate and differentiation of these cells.

## Introduction

*Chlamydia trachomatis* is an obligatory intracellular bacterium. And it is the utmost sexually transmitted infection and the primary reason of bacterial conjunctivitis globally [1]. Understanding how *Chlamydia* interacts with its host is essential for understanding the molecular pathways of infection and development of effective therapeutic strategies. *Chlamydia* has a distinct intracellular life cycle. *C. trachomatis* exhibits a unique intracellular lifecycle in epithelial cells and immune cells such as macrophages. *Chlamydia* enters the host cells via endocytosis and resides in a bacterial-containing vacuole called an inclusion. Inclusion protects *Chlamydia* cells from the host cell’s innate immune mechanisms [2]. Once in the host cells, *Chlamydia* alternates through a biphasic development cycle, transitioning among the infectious elementary body (EB) and the replicate reticulate body [3, 4].

To ensure its survival within the host, *C. trachomatis* has evolved several strategies to manipulate host cell machinery, modulate host cell signaling pathways, and inhibit apoptosis [3]. Additionally, *Chlamydia* is known to subvert innate immune responses, enabling the pathogen to replicate within the host cell. Being a primary site of infection, epithelial cells are considered the main host for chlamydial infection and replication, offer valuable comprehensions into the acute and chronic events of *Chlamydia*l infection and the subsequent host responses. Studying *C. trachomatis* interactions with epithelial cells can reveal the complexities of the infection process, host cell factors crucial for bacterial survival, and potential targets for therapeutic intervention [2].

Mesenchymal Stem Cells (MSCs) have emerged as intriguing models in the field of infection biology because of their unique immunomodulatory properties and regenerative potential [5]. MSCs, traditionally recognized for their capability to differentiate [6] into various cell types, exhibit remarkable interactions with the immune system, making them a subject of interest in infectious disease research [7, 8]. MSCs possess the capacity to migrate to sites of inflammation and infection, responding to signals from damaged tissues and activated immune cells [6]. Once the MSCs are at the site of infection, they can dynamically modulate the immune response through paracrine signaling, secretion of anti-inflammatory factors, and interaction with immune cells [6]. Furthermore, studies suggest that MSCs may be able to directly combat microbial infections [9, 10]. This can involve antimicrobial peptide secretion, phagocytosis, and other direct interactions with pathogens [6, 7, 11]. The precise mechanisms through which MSCs exert their effects on infectious agents are an active area of investigation, with implications for designing innovative therapeutic strategies.

This study aims to explore the influence of *C. trachomatis* direct infection on the pluripotency of bone marrow derived MSCs and to observe potential alterations in or differentiation of the cells following infection. Within this context, we analyzed the expression levels of pluripotency and MSC-specific markers throughout the infection. Further, we determined whether infection of MSCs could directly/indirectly affect the differentiation of the MSCs and to an osteogenic, chondrocytic or adipogenic phenotype. This will not only enhance our comprehension of *Chlamydia* pathogenesis but also makes a valuable contribution to the broader domain of host-pathogen interactions.

## Materials and Methods

### Chemicals and Antibodies

Antibodies used in this study, for immunofluorescence analysis and Western immunoblotting, were: mouse anti-CD44 mouse FITC-conjugated (BD Pharmingen, USA), mouse anti-CD73 APC-conjugated (Biolegend, USA), mouse anti-CD90 PE-conjugated (BD Pharmingen), rabbit anti-Oct4 (Sigma Aldrich, USA), rabbit anti-Sox2 (Chemicon, Japan), rabbit anti-Nanog (Merck), mouse monoclonal anti–*C. trachomatis* hsp60 (Enzo Life Sciences, Germany) and mouse monoclonal anti–β-actin (Sigma-Aldrich), and rabbit polyclonal anti-Chlamydia genus-specific Ab (Milan Analytica AG, Switzerland). Appropriate secondary-labeled Abs were purchased from Jackson ImmunoResearch Laboratories for immunofluorescence and from Amersham Biosciences for Western blot analyses.

### Cell Culture

Mesenchymal stem cells were derived from bone marrow (BM-MSCs) (Lonza PT-2501). The mesenchymal stem cells were cultured in 75 cm² and 25 cm² flasks (Techno Plastic Products AG (TPP), Germany) using MSC growth BulletKitTM medium (Lonza PT-3001). Untreated cells were maintained in a HeraCell 150 incubator (Thermo Fisher Scientific, USA) at 37°C and 5% CO_2_. The medium was changed every 2-3 days, and cells reached high confluency before splitting. During splitting, the MSCs were rinsed with D-PBS (Gibco, Germany) and detached using 0.5% trypsin with 5.3 mM EDTA (Gibco). Microscopic images were captured using an Olympus IX50 light microscope equipped with a color digital camera from Scion Corporation. Centrifugations were carried out using an Eppendorf 5415 R centrifuge.

### *Chlamydia* Infection

*C. trachomatis* Lymphogranuloma venereum (LGV) serovar L2 stocks were regularly cultured in HeLa cells cultured in RPMI-1640 medium containing glutamine and 5% fetal bovine serum (FBS). The procedures for culturing *Chlamydia*, preparation of elementary body (EB) stock, and the determination of inclusion forming units (IFU)/ml were performed following previously established protocols (Al-Zeer *et al*., 2013). For infection studies, MSCs were seeded into 6-well plates and allowed to adhere overnight. Subsequently, host cells were infected with *C. trachomatis* at a multiplicity of infection (MOI) of 1, then incubated at 35°C with 5% CO_2_. Two hours after post-infection (h.p.i), cells were washed and replenished with a new medium. The cell cultures were then maintained under the conditions for 24 or 48 h.

### Fluorescence Confocal Microscopy

Around 2×10^5^ MSCs were seeded in 12-well-plates onto coverslips then incubated at 37°C and 5% CO_2_. Next, cells were infected with *C. trachomatis* at a MOI of 1 and incubated at 35°C and 5% CO_2_. Two h.p.i, cells were washed, supplied with new medium, and incubated as before. At specified time intervals, fixed cells using 4% paraformaldehyde (25 min at RT), were permeabilized with 0.3% Triton-100x followed by blocking with 2.5% BSA in PBS. Sequentially, cells were incubated for 1 h with primary and secondary antibodies at RT. Then, coverslips were mounted onto glass slides using mowiol and examined by Leica TCS-SP laser scanning confocal microscope equipped with a krypton/argon laser. Images were processed using Adobe Photoshop 6.0 (Adobe Systems) and Microsoft Power-Point.

### Protein Extraction and Western Blotting

Cells were lysed with SDS lysis buffer (100mM Tris/HCl, pH 6.8, 4% SDS, 20% glycerol, 0.02% bromophenol blue, 200nM dithiothreitol), directly. Cell lysates were boiled at 95°C for 10min. Equal amounts of protein were separated using SDS–polyacrylamide gel electrophoresis and immunoblotted as described previously [12].

### Flow Cytometry

Infected cells with *C. trachomatis* or control uninfected cells were washed with PBS, trypsinized, and centrifuged at 100X G. Then, cells were suspended in warm 4% paraformaldehyde solution in PBS. Cells were washed then incubated for 30 min with labeled antibodies specific for CD44-FITC (1:100), CD73-APC (1:100) and CD90-PE (1:100) at 4°C. Labeled cells were analyzed using a fluorescent associated cell sorter (FACS Calibur, Germany).

### RNA Isolation and qPCR

Using the RNeasy Kit (Qiagen, USA), total RNA from different samples was isolated according to the manufacturer’s protocol. The OneStep RT-PCR Kit (Qiagen) was used for cDNA preparation and amplification. Supplement 1 shows a list of nucleotides used in this study.

### Alkaline Phosphatase Detection

Detection and quantification of alkaline phosphatase was carried out using (StemTAG, Cell Biolabs, USA). Equal amounts of protein were used for alkaline phosphatase detections. The StemTAG detection kit enzymatically degrades pNPP to the yellow pNP product, which can be detected at 405 nm. Eventually, absorbance at 405 nm was measured using a plate reader.

### Statistical Analysis

Statistical analysis was carried out by pairwise comparisons using unpaired, 2-tailed t test in Microsoft Excel.

## Results

### *Chlamydia* Infection Induces the Degradation of Stemness Markers in MSCs

**Fig. 1:**
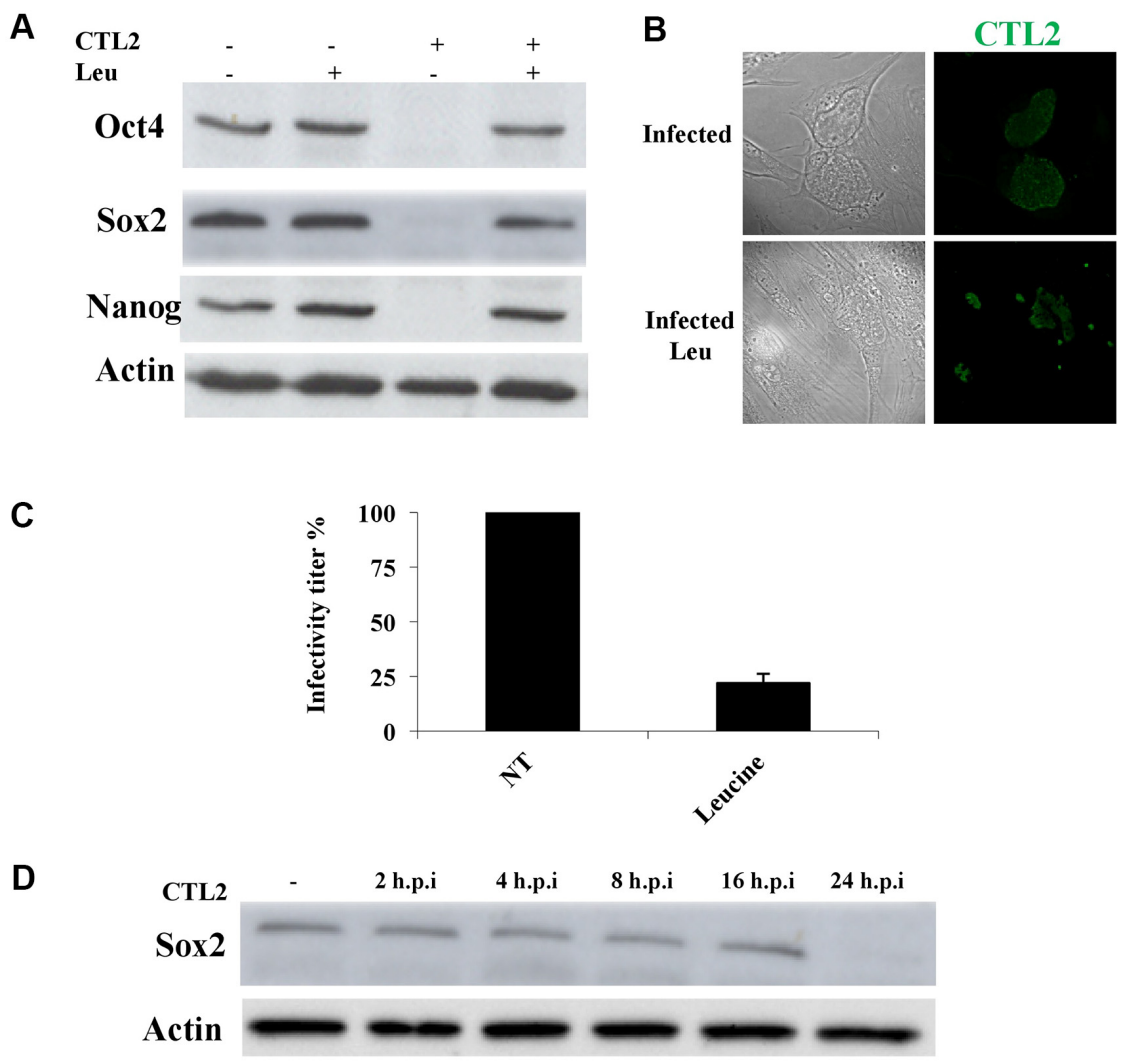
Oct4, Sox2, and Nanog protein expressions are reduced upon *Chlamydia* infection in MSCs. MSCs were infected with *C. trachomatis* (MOI = 1) or left without infection (control). (**A**) Anti- Oct4, Sox2, and Nanog immunoblot analysis of total cell lysates from infected (48 h.p.i) and uninfected cells. All stemness markers are reduced to an undetectable level 48 h.p.i. (**B**) Immunofluorescence images of *Chlamydia* infected MSCs (MOI = 1), treated with 10 mM leucine or left untreated as control, then fixed and labeled with antibody against *C. trachomatis* hsp60 (green). Leucine treatment resulted in reduction of chlamydial inclusion size. Scale bar, 30 μm. (**C**) The yield of *C. trachomatis* infectious progeny reduced significantly upon leucine treatment. Infectivity was calculated as IFU per milliliters and expressed as percentage of control cells. Results shown are the mean values of three independent experiments. Error bars: ± SD. *p < 0.05. (**D**) Anti-Sox2 immunoblot analysis of total cell lysates from infected MSCs between 2-24 h.p.i. Sox2 expression is decreased by 24 h.p.i. β-actin was used as control.

To investigate the effect of chlamydial infection on the expression of stemness markers, we used human MSCs as a model. MSCs, known for their remarkable migratory capability towards sites of infection or tissue injury, are crucial for wound healing. The recruitment of MSCs to the infection sites is facilitated by the release of pro-inflammatory cytokines and chemokines. At the site of infection, MSCs exhibit the potential to modulate the local immune response, facilitate tissue repair, or sometimes become infected themselves. Due to the broad spectrum of natural host infection tropism displayed by *Chlamydia*ceae, various infection models have been used to explore interactions between the host and *Chlamydia*. Notably, one of these models involves primary human MSCs, as outlined by Abu Lubad *et al*., in 2014 [13]. Our prior work has successfully established and utilized MSCs as an infection model, as an alternative model to the commonly utilized tumorigenic cell lines. In the current study, western immunoblotting of MSCs infected with *C. trachomatis* L2 demonstrated a significant decrease in total cellular stemness protein markers (Oct4, Nanog, and Sox2) to nearly undetectable levels at 48 h post-infection (h.p.i) (Fig. 1A). Treatment of infected cells with leucine (5 mM) from 2h.p.i was not only adequate to inhibit inclusion growth, as observed previously (Braun *et al*., 2008), but also effective in rescuing the stemness markers from degradation (Fig. 1A and 1B). Importantly, the viability of host cells remained unaffected by high concentrations of leucine, as assessed by XXT (data not shown). Notably, like the effect of leucine treatment on inclusion size (Fig. 1B), there was a significant reduction in the infectious progeny upon leucine treatment, indicating an inhibition in the normal developmental cycle of *Chlamydia* (Fig. 1C). To delineate the time-course of stemness markers downregulation, total cell lysates from infected cells were collected at 2, 4, 8, 16, and 24 h.p.i. Sox2 protein levels exhibited no changes at 2, 4, 8, and 16 h.p.i but reached almost undetectable levels at 24 h.p.i.(Fig. 1D).

**Fig. 2:**
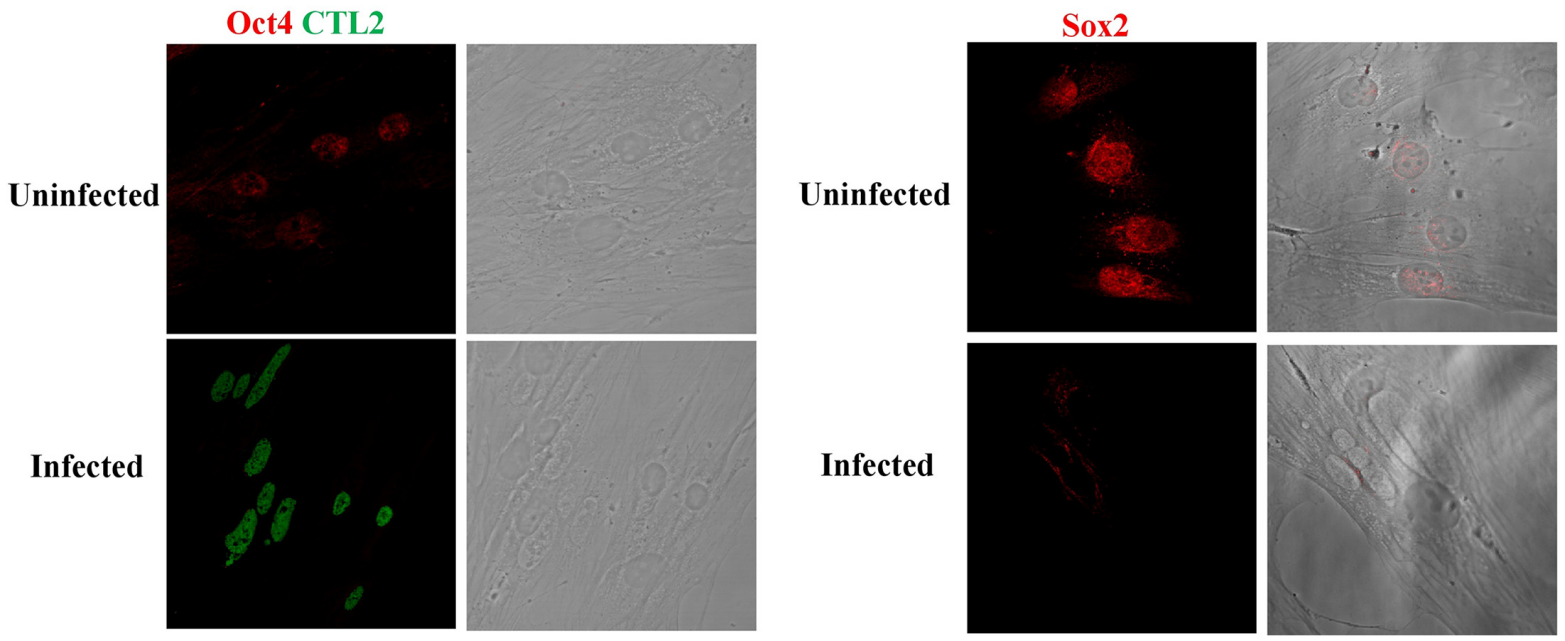
Oct4 and Sox2 protein expressions are reduced upon *C. trachomatis* infection in MSCs. Immunofluorescence micrographs of *C. trachomatis* infected MSCs (MOI = 1), labeled with antibody against *C. trachomatis* hsp60 (green), Oct4 (red), or Sox2 (red). Scale bar, 30 μm. Infection caused a decrease in expression of Oct4 and Sox2 in infected cells by 48 h.p.i. Scale bar, 30 μm.

To further confirm the western blotting results, we used immunofluorescence microscopy. Firstly, primary MSCs were infected with *Chlamydia* at multiplicity of infection (MOI)=1. Then, the samples were fixed prior to immunofluorescence using antibodies against Sox2, Oct4, and *Chlamydia* (Fig. 2). Microscopic analysis corroborated those non-infected cells exhibited noticeable signals for Oct4 and Sox2 in the nucleus. However, these signals displayed a significant reduction upon infection with *C. trachomatis*, consistent with our observations using Western blotting. In summary, our data collectively suggests that acute infection with *Chlamydia* leads to the degradation of various stemness markers within MSCs.

### MSCs-Specific Surface Markers Are Not Altered upon Infection with *Chlamydia*

**Fig. 3:**
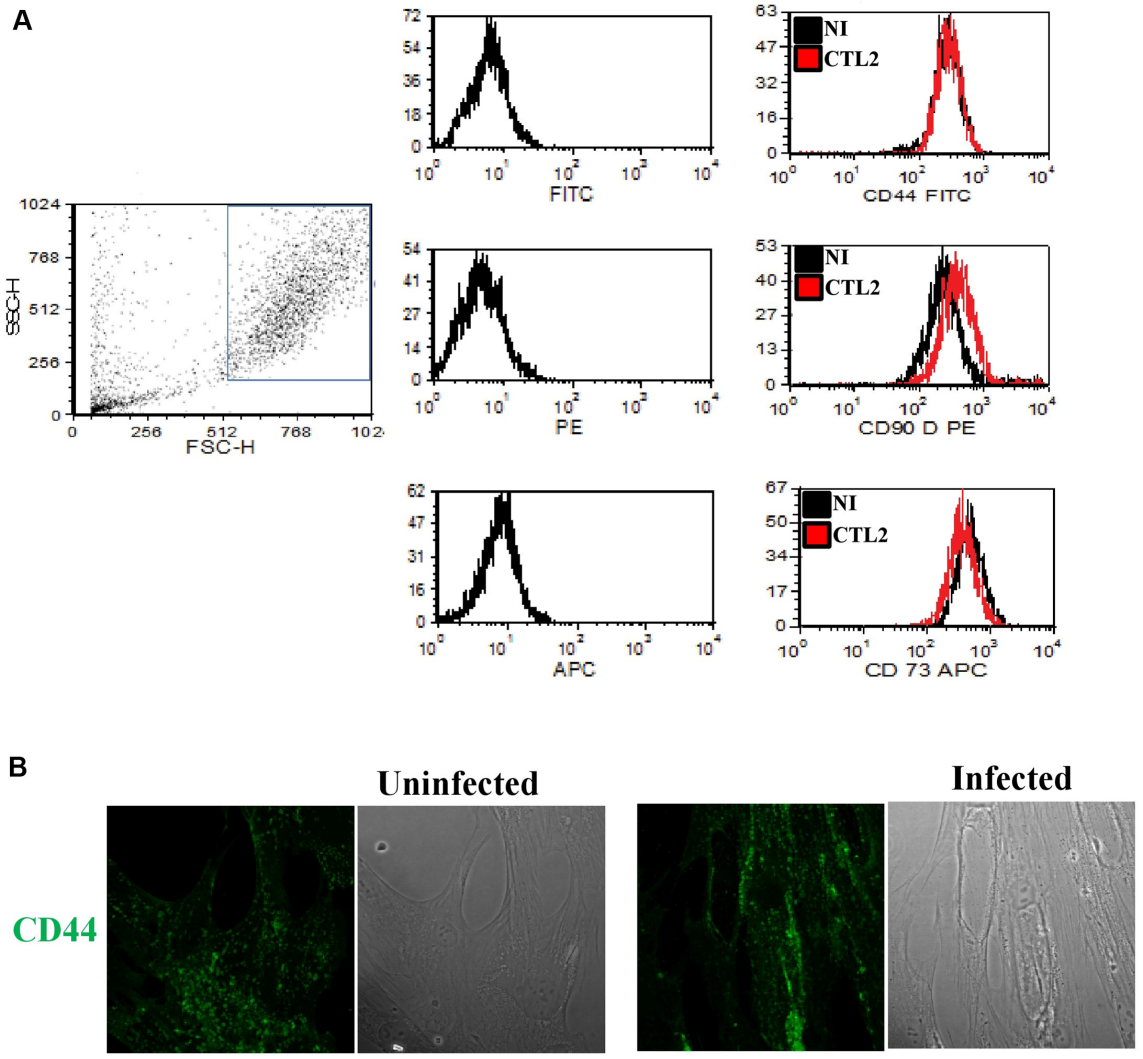
MSCs specific surface markers expression is not altered after infection with *C. trachomatis*. (**A**) After infection with *C. trachomatis* (MOI 1), cells were analyzed for the surface markers CD44, CD73 and CD90 by fluorescent staining and subsequent analysis by flow cytometry. The histograms show infected (red) or uninfected (black) MSCs analyzed for the indicated surface markers (CD44-FITC, CD90-PE, and CD73-APC). Levels of CD44, CD73, and CD90 remain unaffected upon infection with *Chlamydia*. (**B**) Immunofluorescence micrographs of MSCs (infected and control) labeled with antibody against CD44 (green). Infection did not result in changes in expression of CD44. Scale bar, 30 μm.

The decline in pluripotency markers following chlamydial infection could hold a pivotal role in influencing cell fate and the differentiation process in infected cells. Hence, a comparative analysis between uninfected MSCs and cells infected with *Chlamydia* for 48 h.p.i was conducted to discern alterations in MSC-specific surface markers. Remarkably, the cells were analyzed for various surface markers including CD44, CD73, and CD90, employing fluorescent staining followed by analysis using flow cytometry. Interestingly, while CD73 and CD44 showed no discernible changes in infected cells when juxtaposed with control uninfected cells, a marginal elevation in CD90 expression was observed in infected MSCs. Nevertheless, this effect did not reach statistical significance (Fig. 3A). To substantiate these results, we conducted an analysis of CD44 protein expression through immunofluorescence. Similarly, *Chlamydia* infection did not lead to a reduction in CD44 protein expression (Fig. 3B). These findings strongly indicate that the infection did not exert regulatory effects on MSC-specific surface markers, in contrast to the observed impact on stemness markers.

### Chlamydia Infection Triggers Alterations in Various Differentiation Markers within MSCs

**Fig. 4:**
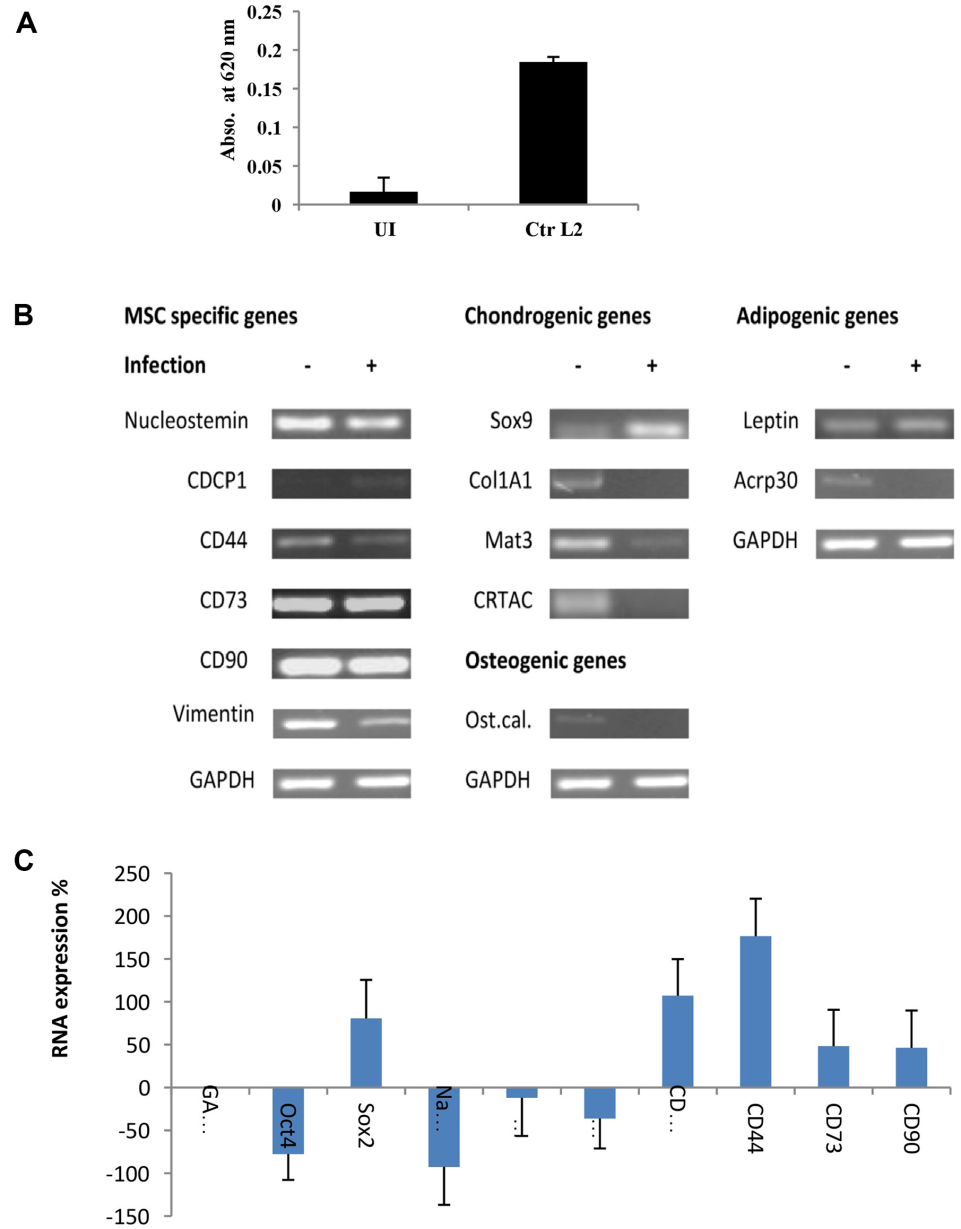
Alkaline phosphatase and differentiation markers are altered in MSCs upon infection with *Chlamydia*. (**A**) Alkaline phosphatase activity in infected cells is increased in comparison with uninfected cells control. (**B**) Touchdown PCR results for the detection of RNA expression of cell specific genes. These genes are specific for MSCs and different differentiation stages including chondrogenesis, osteogenesis or adipogenesis. A GAPDH control shows that equal amount of RNA was applied. (**C**) Comparative analysis of RNA expression between infected and uninfected MSCs at 48 h.p.i. Data represent triplicate data. Error bars indicate standard deviation.

Alkaline phosphatase (AP), an enzyme crucial in the hydrolysis of phosphate esters, serves as a key indicator linked to the stemness and differentiation capacity of MSCs. Particularly, it is often associated with osteogenic and chondrogenic differentiation [14]. Hence, we examined the levels of AP in cells infected with *C. trachomatis*. As depicted in Fig. 4A, *Chlamydia* infection led to a significant increase in AP activity compared to control uninfected cells, providing further evidence that aligns with our observation of the decline in intracellular stemness markers.

Subsequently, a touchdown PCR was conducted using cell-specific primers to, qualitatively, compare the RNA expression of different differentiation markers between infected and uninfected control cells. Notably, among the tested MSCs-specific genes, CD44 and vimentin demonstrated downregulation (Fig. 4B). For CDCP1, an obvious increase in RNA expression was observed upon infection, a result further confirmed through quantitative real-time PCR where CDCP1 exhibited a 100% upregulation (Fig. 4B and 4C). Furthermore, *Chlamydia*l infection notably induced the expression of early chondrogenesis gene Sox9. In uninfected cells, robust expression of Matrilin-3 and CRTAC was evident but lost upon infection. Desmin, a marker for myogenesis, did not yield a detectable PCR product. Additionally, the osteogenic marker IBSP remained undetectable, while osteocalcin and Col1A1 exhibited higher expression in uninfected MSCs compared to infected cells. Interestingly, the expression of Col1A2, another osteogenic marker, remained unchanged upon infection (Fig. 4B). The RNA levels of adipogenic genes leptin and FABP4 exhibited no significant changes, while Acrp30 expression declined following infection. Proliferator-Activated Receptor Gamma 2 (PPARγ2) and Fatty Acid -Transport Protein 1 (FATP1), and C/EBPα were undetectable in both uninfected and infected MSCs. Taken together, our data indicates that infection with *Chlamydia* induces AP activity and changes in the expression of various MSCs markers including the increase in Sox9 level in infected cells. These changes resemble changes in MSCs undergo differentiation toward chondrogenesis, possibly.

## Discussion

*Chlamydia* is characterized by their capability to infect epithelial cells, macrophages and other host cells including MSCs. Since MSCs lack immortalization or tumorigenic properties, they offer a model akin to primary cells. Given their natural distribution across various tissues and their pivotal roles in immune function, MSCs are susceptible to targeting by bacteria and viruses, potentially leading to various diseases. Interestingly, MSCs supported the life cycle of *Chlamydia*, completely, including entry, replication, and production of infectious chlamydial progeny with comparable efficiency with the infection of human cell lines [13, 15, 16]. MSCs play a crucial role in combating infectious diseases by secreting antimicrobial factors to clear pathogens and directly engulfing certain bacteria [9, 17, 18]. Moreover, MSCs mitigate tissue damage during infections by suppressing pro-inflammatory cytokine levels at the site of injury [19, 20]. Furthermore, MSCs facilitate tissue repair by either transforming into the necessary cell types to replace damaged tissue or by releasing signaling molecules that guide tissue regeneration, differentiation, and wound healing processes [21]

MSCs can differentiate into multiple cell lineages including adipocytes, osteoblasts, and chondrocytes under typical differentiation conditions [22]. Since the differentiation of MSCs in course of chlamydial infection remains largely unknown, addressing the behavior of MSCs in infectious environment would better explain this story. It has been demonstrated that infection of MSCs with *C. trachomatis* induces degradation of p53, reduction in iNOS expression, and most recently downregulation of p27 [13, 15, 16]. Interestingly, in our study, *Chlamydia* infection in MSCs induced loss of stemness markers, a novel phenotype which has not been shown previously for bacterial infection. This degradation of stemness markers in MSCs upon infection with *Chlamydia* might happen through various pathways, particularly involving the outer membrane proteins (OMPs) of *Chlamydia*. These proteins are thought to induce inflammatory responses and activate signaling pathways such as mitogen-activated protein kinase (MAPK) pathways in response to infection that promote differentiation and reduce stemness [23, 24]. Additionally, it has been shown that *Chlamydia* stimulates the proteasomal degradation of the tumor suppressor protein p53, which is essential for maintaining stemness [15]. Furthermore, *Chlamydia* may secrete factors like heat shock proteins and proteases that likely disrupt MSC functions directly [25]. Collectively, these mechanisms suggest that *C. trachomatis* infection significantly impacts stemness maintenance in MSCs, indicating that this field of research is very interesting and needs further investigation.

The loss of Oct4, Nanog, and Sox2 expression in *Chlamydia*-infected MSCs may lead to alterations in the differentiation, regenerative, and tissue repair abilities of MSCs. When these stemness markers are downregulated during *Chlamydia* infection, MSCs may show reduced capacity to differentiate into various cell lineages, such as adipocytes, osteoblasts, and chondrocytes. Several model infections have been shown to prompt MSCs differentiation. Na *et al*. reported that MSCs infected with adenovirus-36 undergo adipogenesis, evidenced by the formation of lipid droplets within 48 h post-infection [26]. Additionally, they observed upregulation of several genes involved in adipogenesis following adenoviral infection of MSCs. Based on these findings, Na *et al*., proposed that this differentiation mechanism could elucidate the association between adenoviruses and obesity formation. Similarly, Gibellini *et al*. demonstrated that infection with HIV-1 directs MSCs towards an increased adipogenic pathway and reduced endotheliogenic differentiation [27]. A recent study revealed that HIV infection can modify the viability and immunological profile of Adipose-Tissue-Derived MSCs, potentially impacting MSC-based cell therapies [28].

A reduction in alkaline phosphatase activity is often associated with a loss of stemness in MSCs. This decline in alkaline phosphatase activity may coincide with alterations in the expression of other stemness markers and a diminished capacity for self-renewal and differentiation into multiple cell lineages [29]. Surprisingly, alkaline phosphatase activity was not decreased upon infection with *Chlamydia* in MSCs. Instead, a slight increase in the activity of this enzyme was observed. It has been shown that high alkaline phosphatase activity in MSCs normally indicates a state of active osteogenic differentiation [30]. Alkaline phosphatase is an enzyme intricate in bone mineralization and is upregulated during osteogenesis [30]. Therefore, the increased levels of alkaline phosphatase activity in *Chlamydia*-infected MSCs may suggest that they are committing to the osteogenic lineage and undergoing differentiation into osteoblasts, which are bone-forming cells. However, the loss of stemness in *Chlamydia*-infected MSCs, and the ability to maintain acute infection for up to 48-72 h, may result in impaired differentiation potential toward complete osteogenesis, which requires further investigation. While some bacteria might influence the stemness and differentiation capacity of MSCs, the body of research remains limited, particularly regarding the distinct impacts of obligate intracellular versus extracellular bacteria. For example, extracellular bacteria like *Staphylococcus aureus* can also modulate MSCs function through various metabolites and signaling molecules [31-33] . It has been shown that *S. aureus* might inhibit osteogenic gene expression and increase levels of miR-29b-3p, which negatively regulates differentiation [23, 33]. While α-hemolysin reduces osteogenesis [31], both lipoteichoic acid and Staphylococcal enterotoxin C2 promote it by enhancing autophagy and boosting osteogenic markers [32].

It has been established that during chondrogenesis and osteogenesis, alkaline phosphatase activity is initially increased and subsequently declines [34]. This pattern exemplifies a non-continuous gene regulation phenomenon in the differentiation process. Consequently, genes associated with differentiation are categorized into early, mid, and late stages [34]. Interestingly, there was a notable reduction in RNA levels of vimentin, indicating a loss of a significant characteristic in MSC [35]. Similarly, desmin, a gene associated with myogenesis, was undetectable, and the adipogenesis-related genes examined in this study remained unchanged following infection. In contrast, there were significant alterations in the RNA levels of genes associated with chondrogenesis. For instance, the Sox9 gene, known as an early-stage chondrogenesis factor, exhibited up-regulation during chondrogenesis [34]. However, the high expression of Matrilin-3 and CRTAC in uninfected cells, which diminishes after infection, contradicts the process of chondrogenesis. A possible explanation for the elevated expression of Matrilin-3 could be the origin of these cells from bone marrow. Additionally, it's important to note that transcriptional data alone are insufficient to confirm the presence of these markers at the protein level; thus, Western blot experiments are necessary to clarify this. The same applies to osteogenesis-related genes such as osteocalcin and Collagen Type I Alpha 1 (Col1A1), which also show down-regulation after infection.

The increase in Sox9 expression, known for its role in chondrogenesis, hints at an enhanced capacity for MSCs to transform into cartilage-forming cells. Conversely, the diminished expression of CRTAC1 and matrilin, both associated with components of the extracellular matrix, suggests a departure from specific structural elements. Moreover, the simultaneous decrease in adiponectin and osteocalcin implies a suppression of adipogenic and osteogenic pathways. Furthermore, CD44 up-regulation could also be an indication for osteogenesis or chondrogenesis. However, even an altered expression of surface markers would not be sufficient to characterize a distinct cell.

Overall, these changes collectively suggest a significant alteration in the differentiation potential of MSCs, potentially favoring chondrogenic differentiation while suppressing adipogenic and osteogenic pathways. However, it's important to note that alterations in surface markers alone may not be sufficient to fully characterize the distinct cell phenotype, as cellular behavior is governed by a complex interplay of various factors beyond gene expression. Further functional assays and characterization would be needed to fully understand the implications of these molecular changes on MSC behavior and differentiation potential.

This complex regulatory pattern suggests that MSCs may be directed towards chondrogenic differentiation, indicated by the up regulation of CD44 and Sox9, and increased expression of alkaline phosphatase. Concurrently, there appears to be a suppression of adipogenic and osteogenic differentiation pathways. The altered expression of these markers highlights a nuanced and coordinated adjustment in the differentiation potential of MSCs upon infection with *C. trachomatis*. To confirm whether differentiation is indeed taking place and to pinpoint the specific cell lineage undergoing development, extending the infection time is essential. Ideally, establishing a persistent *C. trachomatis* infection would offer the most comprehensive insights. The interaction between chlamydia and MSCs has been established, shedding light on a potential novel approach to comprehending the intricacies of chlamydial infections. This encompasses actions such as apoptosis inhibition, metabolic reprogramming, suppression of NO synthase, and induction of polyamine synthesis [13, 15, 36]. Considering the significant therapeutic potential of MSCs in combating various infections globally, it becomes imperative to explore their antimicrobial efficacy against both Gram-positive and Gram-negative organisms. These inherent properties of MSCs underscore their pivotal role in therapeutic development. However, a deeper understanding of MSCs' interactions with microorganisms is necessary. Hence, we delineate key areas warranting further investigation regarding MSCs. This includes exploring stemness markers and the differentiation potential of MSCs upon infection. Moreover, there is a need for additional research to elucidate their mechanism of action and establish diverse infection models to ascertain the impact on stemness loss, ultimately striving for consensus in addressing infectious diseases.

## Figures and Tables

**Table 1:** Primer sets used in the present study.

Primer name	Orientation	Sequence 5’ 3’	Trancript ID
Oct4	Sense	GAGTGAGAGGCAACCTGGAG	NM_002701.4
Antisense	GCCGGTTACAGAACCACACT		
SOX2	Sense	CATCACCCACAGCAAATGAC	NM_003106.3
Antisense	GCAAACTTCCTGCAAAGCTC		
Nanog	Sense	TTCCTTCCTCCATGGATCTG	NM_024865.2
Antisense	TCTGCTGGAGGCTGAGGTAT		
p21	Sense	ACTTCGACTTTGTCACCGAG	NM_000389.4
Antisense	TCCTCTTGGAGAAGATCAGC		
p53	Sense	CCTCCTCAGCATCTTATCCGA	NM_000546.4
Antisense	TGGTACAGTCAGAGCCAACCTC		
Vimentin	Sense	ACGCCATCAACACCGAGTTC	NM_003380.3
Antisense	TCGTTGGTTAGCTGGTCCAC		
Nucleostemin	Sense	ATTGCCAACAGTGGTGTTCA	NM_206825.1
Antisense	GTTTCCAAAGGCCCTCTTTC		
CDCP1	Sense	GTTCAAGCTGGAGGACAAGC	NM_178181.1
Antisense	CTCTTGCTGGGTCCAGAGTC		
PPARγ2	Sense	TCCATGCTGTTATGGGTGAA	NM_015869.4
Antisense	TCAAAGGAGTGGGAGTGGTC		
C/EBPα	Sense	AACCTTGTGCCTTGGAAATG	NM_004364.3
Antisense	CCCTATGTTTCCACCCCTTT		
FABP4	Sense	AACCTTAGATGGGGGTGTCC	NM_001442.2
Antisense	TGGTTGATTTTCCATCCCAT		
IBSP	Sense	GACTGCCAGAGGAAGCAATC	NM_004967.3
Antisense	ACCCTGTATACCCTGTGCCA		
Osteocalcin	Sense	GGTGCAGAGTCCAGCAAAGG	NM_199173.4
Antisense	GCCGATAGGCCTCCTGAAAG		
Col1A1	Sense	CACTGGTGCTAAGGGAGAGC	NM_000088.3
Antisense	CTCCAGCCTCTCCATCTTTG		
Col1A2	Sense	GATATTGCACCTTTGGACAT	NM_000089.3
Antisense	CCCACAATTTAAGCAAGAAG		
SOX9	Sense	CACACAGCTCACTCGACCTTG	NM_000346.3
Antisense	TTCGGTTATTTTTAGGATCATCTCG		
Aggrecan	Sense	GAAAGGTGTTGTGTTCCACT	NM_001135.3
Antisense	GTCATAGGTCTCGTTGGTGT		
Desmin	Sense	ATACCGACACCAGATCCAGTCC	NM_001927.3
Antisense	TCCCTCATCTGCCTCATCAAGG		
GAPDH	Sense	GGTATCGTGGAAGGACTCATGAC	NM_002046
Antisense	ATGCCAGTGAGCTTCCCTTCAG		
CD44	Sense	AGATGGAGAAAGCTCTGAGC	NM_000610.3
Antisense	GGTAATTGGTCCATCAAAGGC		
CD73	Sense	CGCAACAATGGCACAATTAC	NM_002526.3
Antisense	CTCGACACTTGGTGCAAAGA		
CD90	Sense	CTAGTGGACCAGAGCCTTCG	NM_006288.3
Antisense	GCACGTGCTTCTTTGTCTCA		
