# Corrigendum to: Loss in Pluripotency Markers in Mesenchymal Stem Cells upon Infection with *Chlamydia trachomatis*

**DOI:** 10.4014/jmb.2025.3505.C01

**Published:** 2025-06-05

**Authors:** Munir A. Al-Zeer, Daniel Lauster, Mohammad Abu Lubad

**Affiliations:** 1 Department of Biological Sciences, School of Science, The University of Jordan, Amman 11942, Jordan; 2 Microbiology and Immunology Department, Faculty of Medicine, Mutah University, Al-Karak, Jordan; 3 Freie Universität Berlin, Institute of Pharmacy, Biopharmaceuticals, Kelchstr. 31, 12169 Berlin, Germany

In the article titled “Loss in Pluripotency Markers in Mesenchymal Stem Cells upon Infection with Chlamydia trachomatis, by Munir A. Al-Zeer^1^ and Mohammad Abu Lubad^2^, we would like to address an authorship omission that occurred during the submission process. Dr. Daniel Lauster was inadvertently omitted from the original author list. Dr. Lauster contributed to the experimental design and analysis, and his inclusion as a coauthor is fully warranted.

The corrected author list and affiliations are as follows:

Munir A. Al-Zeer^1*#^, Daniel Lauster^3#^, Mohammad Abu Lubad^2^

^1^Department of Biological Sciences, School of Science, The University of Jordan, Amman 11942, Jordan

^2^Microbiology and Immunology Department, Faculty of Medicine, Mutah University, Al-Karak, Jordan

^3^Freie Universität Berlin, Institute of Pharmacy, Biopharmaceuticals, Kelchstr. 31, 12169 Berlin, Germany

We acknowledge this correction and confirm that it does not affect the results, interpretations, or conclusions of the article.

# equal contribution

